# Effects of combined cytotoxic T-lymphocyte antigen 4 and programed death 1 ligand-receptor blockade on interferon-gamma production in bovine leukemia virus-infected cattle

**DOI:** 10.14202/vetworld.2024.1672-1679

**Published:** 2024-08-03

**Authors:** Sergey Borovikov, Kanat Tursunov, Zhansaya Adish, Laura Tokhtarova, Kanatbek Mukantayev

**Affiliations:** 1 Laboratory of Immunochemistry and Immunobiotechnology, National Center for Biotechnology, Astana, Kazakhstan; 2 Department of Microbiology and Biotechnology, Faculty of Veterinary and Animal Husbandry Technology, S. Seifullin Kazakh Agrotechnical Research University, Astana, Kazakhstan; 3 Department of Natural Sciences, L.N. Gumilyov Eurasian National University, Astana, Kazakhstan

**Keywords:** bovine, bovine leukemia virus, cytotoxic T-lymphocyte-associated antigen 4, interferon-gamma, programmed death ligand 1

## Abstract

**Background and Aim:**

In chronic viral infections, cytotoxic T-lymphocyte-associated antigen 4 (CTLA-4) and programmed death ligand 1 (PD-L1) significantly suppress immune responses. The CTLA-4 receptor abundance in regulatory T cells showed a positive association with viral load and a negative association with interferon-gamma (IFN-γ) production in bovine leukemia virus (BLV)-infected cattle. Blocking this receptor boosted IFN-γ production, recovering immune response against this illness. In human cancer patients, not everyone responded positively to non-immunotherapy using CTLA-4 receptor antibodies. The present study analyzed the synergistic effects of CTLA-4 and PD-L1 receptor blockade on IFN-γ production in BLV+ cattle in vitro.

**Materials and Methods:**

The genes for bovine CTLA-4 and PD-L1 were artificially produced. The amino acid sequences of the extracellular receptor domains were sourced from the National Center for Biotechnology Information PubMed database. The western blotting and liquid chromatography with tandem mass spectrometry (LC-MS/MS) techniques were employed for the characterization of recombinant CTLA-4 (rCTLA-4) and recombinant PD-L1 (rPD-L1) proteins. The immunoinhibitory effects of recombinant proteins in *Staphylococcus enterotoxin* B (SEB)-stimulated cattle peripheral blood mononuclear cells (PBMCs) were investigated. Enzyme-linked immunosorbent assay (ELISA) was used to analyze monoclonal antibodies against rCTLA-4 and rPD-L1. Antibodies generated from peripheral blood mononuclear cells of healthy and BLV-seropositive cows were employed to evaluate their blocking capabilities.

**Results:**

The resulting recombinant proteins specifically reacted with commercial homogeneous monoclonal antibodies (mAbs) using ELISA and anti-His-tag mAbs using western blotting. Analysis of the proteins using LC-MS/MS revealed correspondence with the sequences of rCTLA-4 and rPD-L1 located in the Mascot database. rCTLA-4 and rPD-L1 proteins inhibited IFN-γ production in bovine PBMCs of activated SEB. When PBMCs from cows were cultured with activated SEB containing rCTLA-4 and rPD-L1, the mAbs increased IFN-γ production in PBMCs. The combined cultivation of mAbs and PBMCs from BLV+ cattle enhanced IFN-γ production in the cells.

**Conclusion:**

These findings suggest that the combined blockade of bovine CTLA-4 and PD-L1 receptors can be used as a therapy for bovine leukemia. However, it was shown that a single PBMC sample from a BLV-positive donor did not amplify the synergistic effect. Therefore, it is necessary to perform further studies on a larger population and assessing a wider range of cytokines.

## Introduction

Immune checkpoint blockade is a promising strategy for treating various chronic infections. This strategy is particularly relevant for preventing the spread of chronic infections, such as paratuberculosis, brucellosis, and bovine leukemia. Bovine leukemia virus (BLV) persistently infects CD+ B cells, causing lymphocytosis in 20%–30% of BLV-infected animals [1, 2]. During BLV infection, a decrease in the cellular immune response plays an important role in the progression of the disease and the development of lymphomas in 2%–3% of cows. We observed a positive correlation between viral load and transforming growth factor beta (TGF-β) production by regulatory T cells. In addition, the expression of interferon-gamma (IFN-γ) and tumor necrosis factor-alpha (TNF-α) produced by CD4+ T cells decreased, leading to the suppression of natural killer cells [3]. Immune system dysfunction associated with CD4+CD25 high Foxp3+ and CD4+ T-cell counts contributes to the progression of BLV infections. This dysfunction of the immune system contributes to organ damage and the development of opportunistic infections, such as *Mycobacterium avium* (paratuberculosis subspecies), which causes Johne’s disease [4–7].

The molecular mechanisms underlying the influence of BLV on the host are complex and involve the programmed cell death 1 (PD-1), programmed death ligand 1 (PD-L1), and cytotoxic T-lymphocyte-associated antigen 4 (CTLA-4) signaling pathways, which collectively suppress BLV-specific Type 1 T helper responses. Other immune inhibitors of BLV infection are known, such as T-cell immunoglobulin domain and mucin domain-3 [8]. CTLA-4 is notably upregulated in chronic infections and malignancies, contributing to host immune dysfunction. Expression of this protein is closely associated with disease progression in BLV-infected cattle. Notably, in animals with persistent lymphocytosis, the concentrations of CTLA-4 in CD4+ and CD25+ T cells were significantly higher than those in healthy cows. Blocking CTLA-4 reactivates lymphocyte function and can be a novel therapy for chronic diseases [2, 9]. PD-L1 also plays a substantial role in BLV infection progression. Similar to CTLA-4 blockade, PD-1 and PD-L1 blockade enhances T-cell function and inhibits BLV proliferation. Decreasing the number of PD-1+ T cells by binding to PD-L1 on B cells contributes to the progression of viral infections. Treatment of HIV-infected macaques and lymphocytic choriomeningitis virus-infected mice with PD-L1 or PD-1 antibodies restored multiple functions of previously exhausted T cells, resulting in viral clearance i*n vivo*. Blocking the PD-1/PD-L1 pathway and CTLA-4 have potential clinical applications in treating chronic infections [4, 10]. The synergistic effects of antibodies against bovine PD-L1 and CTLA-4 on immunity in asymptomatic BLV+ cows are noteworthy [11]. Yang *et al*. [12] tested the combination of anifrolumab and ipilimumab in their study, while Preite *et al*. [13] evaluated the combination of other PD-1/PD-L1 and CTLA-4 blockers. However, the effects of the synergistic blockade of bovine CTLA-4 and PD-L1 receptors are poorly understood, and further research is needed. In addition, the therapeutic effects of combined immunotherapy with antibodies against CTLA-4 and PD-L1 have been conducted only in humans [11]. Limited research has been conducted on the complex blockade of CTLA-4 and PD-L1 in cattle.

This study presents data on the synergistic effects of PD-L1 and CTLA-4 antibodies on immune activity in BLV+ cattle. To obtain monoclonal antibodies (mAbs), we used the recombinant extracellular domains of bovine PD-L1 and CTLA-4 expressed in *Escherichia coli*. Prokaryotic expression is the most effective method for obtaining proteins associated with high growth rates of bacterial biomass [14–16]. The recombinant PD-L1 (rPD-L1) and recombinant CTLA-4 (rCTLA-4) proteins were analyzed using enzyme-linked immunosorbent assay (ELISA), western blotting, and LC-MS/MS. The recombinant proteins belong to CTLA-4 and PD-L1 receptors in cattle. The resulting mAbs blocked bovine CTLA-4 and PD-L1 receptors and induced IFN-γ production *in vitro*. In a study of peripheral blood mononuclear cells (PBMCs) collected from healthy and asymptomatic BLV+ cattle, combined blockade of CTLA-4 and PD-L1 receptors resulted in increased IFN-γ production.

This study aimed to determine the synergistic effects of mAbs against CTLA-4 and PD-L1 receptors on the production of IFN-γ in bovine PMBCs.

## Materials and Methods

### Ethical approval

The study was approved by the Ethical Committee of the National Center for Biotechnology, Astana, Republic of Kazakhstan (No.4, 26.08.2021).

### Study period and location

The study was conducted from September 2022 to February 2024 at the National Center for Biotechnology (Astana, Kazakhstan). Whole blood samples were collected from animals on farms and farmsteads in Akmola during routine veterinary examinations. The study involved 12 cows of the black-steppe breed.

### Bacterial strain, plasmid, and antibody

To clone and express genes from the bovine CTLA-4 and PD-L1 receptor extracellular domains, we used *E. coli* DH5α and BL21 (DE3) cells (Novagen, Cambridge, UK). The pET28 vector (Novagen) was used to produce the pET28/rCTLA4-bovine and pET28/PDL1-bovine vectors. Mouse monoclonal antibodies against His-tags (ThermoFisher Scientific, Waltham, MA, USA) were used to identify recombinant proteins. An IFN bovine ELISA Kit (Abcam, Boston, MA, USA) was used to determine the IFN-γ concentration in bovine serum. ID Screen^®^ BLV Competition (IDVet, Grabels, France) was used to detect anti-BLV antibodies in bovine serum samples. Whole blood was collected using a K2 EDTA vacuum tube for PBMC isolation. A BD Vacutainer® Plus Serum was used to obtain serum and test for anti-BLV antibodies.

### Gene synthesis

The bovine PD-L1 and CTLA-4 protein sequences (NP_776722.1 and NP_001156884.1) were acquired from PubMed National Center for Biotechnology Information. Using Vector NTI Advance 11.5.0 (Invitrogen, Madison, WI, USA), DNA was codon-optimized for *E*. coli expression, and the 6-His tag, BamHI, EcoRI, NcoI, and XhoI restriction sites were added. The DNAWorks program (http://helixweb.nih.gov/dnaworks/) was used to design the oligonucleotides. Gene synthesis was performed in accordance with the Dolgova–Stukolova protocol [17]. A Gel Extraction Kit (Qiagen, Germantown, MD, USA) was used to purify and clone the polymerase chain reaction (PCR) output into a pGEM-T plasmid (Novagen). After sequencing, genes were cloned into the pET28 plasmid and transferred to competent *E. coli* BL21 (D3) cells. Transformed *E. coli* cells were analyzed using PCR screening, followed by cryopreservation, cultivation, subsequent isolation, and purification of recombinant proteins.

### Protein purification

The selected transformed *E. coli* BL21 (D3) cells were cultured in 200 mL of LB broth containing kanamycin and incubated at 37°C with stirring at 200 rpm. A 16-h incubation period at 37°C with stirring at 200 rpm was conducted after adding 0.2 mM isopropyl-β-D-1-galactopyranoside (IPTG) in the middle of the log phase. The cells were harvested through centrifugation at 6,000 × *g* for 7 min at 4°C. An ultrasonic disintegrator was used to destroy the cell pellets after being resuspended in a lysis buffer. Once the disintegrating cells were centrifuged, the pellet was resuspended in a buffer containing 1 M urea, incubated at 22°C for 30 min, and then centrifuged at 20,000 × *g* for 30 min. The resulting pellet was resuspended in a buffer containing 8 M urea, and incubation and centrifugation were repeated. Proteins were purified using histogram columns (GE Healthcare, Uppsala, Sweden). Recombinant proteins were refolded as described by Xu *et al*. [18]. The refolded proteins were eluted using a linear imidazole gradient and analyzed using LC-MS/MS and western blotting. The bovine CTLA-4 and bovine PD-L1 ELISA Kits (Kingfisher Biotech, Inc., USA) were used according to the manufacturer’s instructions.

### mAb production

Antibovine rCTLA-4 and rPD-L1 mAbs were established as described by Yokoyama [19]. Hybridomas producing mAbs were selected by screening the culture medium using ELISA. The selected mAbs were purified using a MabTrap kit (ThermoFisher Scientific, Austria). Subclasses were identified using the rapid ELISA Mouse mAb Isotyping Kit (ThermoFisher Scientific). The specificity and affinity of the anti-bovine rCTLA-4 and rPD-L1 mAbs were confirmed using western blotting and ELISA.

The antibody concentration was converted to moles using the following formula:


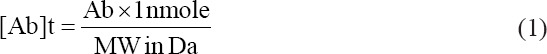


where Ab is mAbs’s concentration (ng) and MW is the molecular weight of Ab (Da).

The constant affinities of the mAbs were determined using the equation provided by Beatty *et al*. [20]:


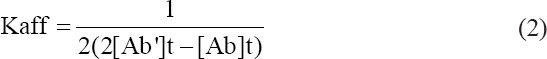


Where: Ab’ is antibody concentration reacted with 5 μg/mL proteins at OD-50; Ab is antibody concentration reacted with 10 μg/mL proteins at OD50.

### CTLA-4 and PD-L1 blockade

PBMCs were isolated from the blood of healthy bovine using LymphoPrep™ (Progen, Heidelberg, Germany) to study the inhibitory effects of the recombinant protein. All cells were grown at a concentration of 1 × 10^6^ cells/well in 24-well plates (Corning Inc., Costar, Salt Lake City, UT, USA) at 37°C with 5% carbon dioxide (CO_2_) and cultured in Roswell Park Memorial Institute medium 1640 (RPMI 1640, Sartorius, Israel) supplemented with 10% heat-inactivated fetal bovine serum (Biological Industries, Israel) and *Staphylococcus enterotoxin* B (SEB) at a concentration of 0.1 μg/mL. Supernatants from cell cultures were used to determine IFN-γ levels using the bovine IFN-γ ELISA Kit (Abcam).

To study the blocking properties of the mAbs, PBMCs from five healthy bovines were cultivated in RPMI 1640 with SEB and the mAbs at a concentration of 20 μg/mL. To synergistically block CTLA-4 and PD-L1, PBMCs were isolated from seven BLV-infected bovine blood samples. The cells were cultured at 37°C with 5% CO_2_ in RPMI 1640 media with 10% heat-inactivated fetal bovine serum. Anti-CTLA-4 (mouse immunoglobulin G [IgG]1), anti-PD-L1 (mouse IgG1), and supernatant from the fetal lamb kidney (FLK) cell culture were used to culture the cells. Mouse IgG was used as a negative control in the presence of heat-inactivated supernatants from FLK cell cultures. The culture medium was analyzed using a bovine IFN-γ ELISA Kit. The PBMC blockade assay and IFN-γ determination were performed as described by Okagawa *et al*. [21].

### Statistical analysis

Data were analyzed and visualized using GraphPad Prism version 9.3.1 (https://www.graphpad.com/). The statistical significance (p < 0.05) of differences between groups was determined using Student’s t-test and one-way analysis of variance (ANOVA) with p values indicated in the corresponding figure captions.

## Results

### Expression and purification of recombinant proteins

**Figure-1:**
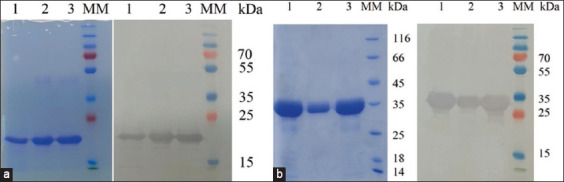
SDS-PAGE and western blot images of purified proteins expressed in the *Escherichia coli* BL21 (DE3) strain. (a) rCTLA-4; (b) rPD-L1. Lanes 1-3 – purified fractions of recombinant proteins; Line 3, molecular weight markers. rCTLA-4=Recombinant cytotoxic T-lymphocyte–associated antigen 4, rPD-L1=Recombinant programmed death ligand 1.

Sequences encoding the extracellular domains of bovine CTLA-4 and PD-L1 receptors were synthesized *de novo*. The resulting genes were cloned into the pET28 plasmid and transformed into BL21 cells. Clones of the transformed cells were screened using PCR for expression activity. Cells were incubated in LB broth at different IPTG concentrations, temperatures, and cultivation times to detect expression activity. The optimal parameters for expressing the recombinant proteins were 0.2 mM IPTG, 37°C, and induction for 16 h. The expressed proteins were characterized using 12% sodium dodecyl-sulfate polyacrylamide gel electrophoresis (SDS-PAGE) and purified using Ni2+-NTA chromatography. Western blotting using mAbs against the His-tagged protein revealed protein bands with similar molecular masses (Figure-1). The electrophoresis results showed proteins with molecular masses of ~20 kDa for rCTLA-4 (Figure-1a) and ~31 kDa for rPD-L1 (Figure-1b).

**Figure-2:**
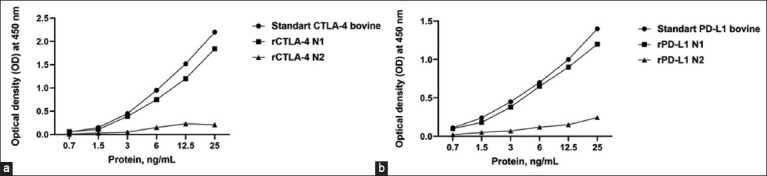
ELISA for assessing the binding of recombinant (a) CTLA-4 and (b) PD-L1 proteins. ELISA=Enzyme-linked immunosorbent assay, CTLA-4=Cytotoxic T-lymphocyte-associated antigen 4, PD-L1=Programmed death ligand 1.

The urea concentration was reduced from 8 M to 0 in the buffer after washing the Histrap columns to ensure proper refolding of the recombinant protein. Proteins were eluted using a linear imidazole gradient (20–500 mmol/L). Positive results were observed when a linear gradient of urea in Histrap columns and dialysis were used to refold the recombinant proteins (Figure-2; rCTLA-4 N1 and rPD-L1 N1). Serial dilution resulted in the refolding of recombinant proteins that did not react with ELISA (Figure-2; rCTLA-4 N2 and rPD-L1 N2).

**Figure-3:**
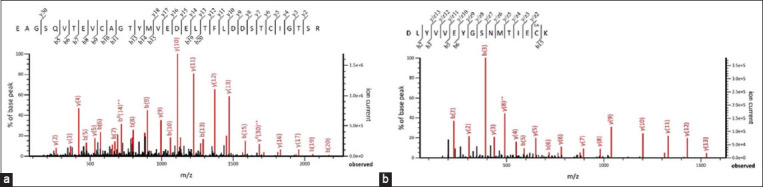
MS/MS spectra of fragmented peptides derived from trypsin-digested bovine (a) rCTLA-4 and (b) rPD-L1. rCTLA-4=Recombinant cytotoxic T-lymphocyte-associated antigen 4, rPD-L1=Recombinant programmed death ligand 1.

The LC-MS/MS spectral data of rCTLA-4 and rPD-L1 bovine ions were analyzed using the Mascot database. The spectral ions corresponded to the bovine CTLA-4 (score 157.9) and PD-L1 (score 375.4) proteins. Figure-3 shows representative MS/MS spectra fragmentation ions of the EAGSQVTEVCAGTYMVEDELTFLDDSTCIGTSR peptide of bovine rCTLA-4 (Figure-3a) and the DLYVVEYGSNVTLECR peptide of bovine rPD-L1 (Figure-3b).

### Generation of monoclonal antibodies to recombinant proteins

**Figure-4:**
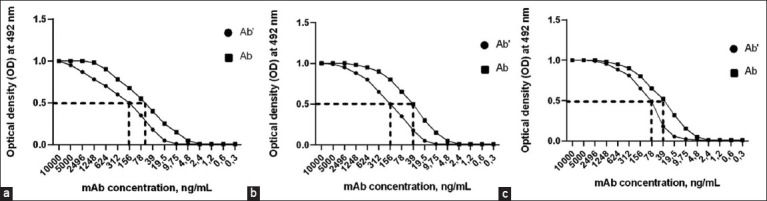
Experimental ELISA curves for mAbs 1c3 (a), 4h8 (b), and 3g2 (c) at different immobilized concentrations of recombinant proteins. ELISA=Enzyme-linked immunosorbent assay, mAbs=Monoclonal antibodies.

Clones 1c3 and 4h8, which produce mAb against bovine CTLA-4, and 3g2, which produce mAb against bovine PD-L1, were selected from the obtained hybridomas. The mAb isotype determination results are shown in Table-1. The mAbs were incubated with recombinant proteins at concentrations of 10 and 5 μg/mL using ELISA to assess constant affinity. Experimental serial mAb dilution curves for two different recombinant protein coating concentrations using three different antibodies are shown in Figure-4. In all three plots, the optical density 50 (OD-50) points shifted to the left as the mAb concentration decreased when different concentrations of the recombinant protein were used. The concentrations of mAbs in nanomoles were calculated using equation (1) and are presented in Table-1.

**Table-1:** Characteristics and affinity constants (Kaff [M^−1^]) of the anti-CTLA4 and anti-PD-L1 mAbs.

mAb	Isotype	Specificity	[Ab’]t, nmole	[Ab]t, nmole	K_aff_
1c3	IgG1	rCTLA-4	0.00104	0.00038	2.9×10^8^-1^−1^
4h8	IgG1	rCTLA-4	0.00104	0.00026	2.7×10^8^-1^−1^
3g2	IgG1	rPD-L1	0.00052	0.00026	6.4×10^8^M^−1^

Ab=Antibody, mAb=Monoclonal antibody, CTLA-4=Cytotoxic T-lymphocyte-associated antigen 4, rCTLA-4=Recombinant cytotoxic T-lymphocyte-associated antigen 4, rPD-L1=Recombinant programmed death ligand 1, IgG=Immunoglobulin G

The mAb concentrations of clone 1c3 binding to 50% of rCTLA-4 were 156 and 58 ng/mL, respectively, and those of clone 4h8 were 156 and 39 ng/mL. The mAb concentrations of clone 3g2 binding to 50% of the rPD-L1 protein were 78 and 39 ng/mL, respectively.

The affinity constant (Kaff) was calculated using equation (2) based on the nmol mAb data presented in Table-1. Kaff (M^−1^) was: for 1c^3^ – 2.9×10^8^M^−1^; 4h8 – 2.7×10^8^M^−1^; and 3g2 – 6.4×10^8^M^−1^.

### IFN-γ production during CTLA-4 and PD-L1 blockade in PBMCs

PBMCs from five healthy cows and seven BLV+ cows with subclinical signs were used. The sera were tested for the presence of specific antibodies to confirm the seropositivity of the cattle. To confirm the effects of the bovine rCTLA-4 and rPD-L1 proteins on immunity, activated SEB PBMCs were cultured with these proteins. A decrease in IFN-γ production in PBMC culture was detected at 0.12 ng/mL.

**Figure-5:**
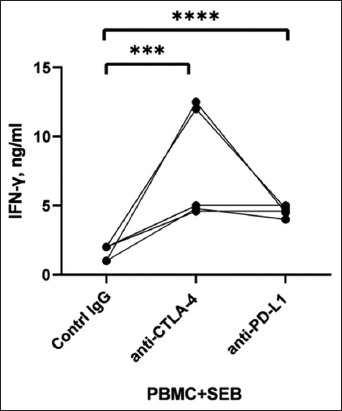
Blocking activity of anti-CTLA-4 and anti-PD-L1 mAbs on PBMCs of activated SEBs. CTLA-4=Cytotoxic T-lymphocyte-associated antigen 4, PD-L1=Programmed death ligand 1, mAb=Monoclonal antibody. PMBC=Peripheral blood mononuclear cell, SEB=*Staphylococcus* enterotoxin B. Student’s t-test was used to compare the statistics between groups. Difference between control immunoglobulin G (IgG) and anti-CTLA-4 antibodies p < 0.0002. Difference between control IgG and anti-PD-L1 antibodies p < 0.0001. Changes were considered statistically significant when the p < 0.001.

To verify the blocking activity of the anti-CTLA-4 and anti-PD-L1 mAbs, PBMCs from five healthy cows were isolated and cultured in the presence of SEB. mAbs 1c3 and 3g2 were then cultured in the supernatant of PBMCs from cows for 7 days [2]. Culture of activated PBMCs with 1c3 and 3g2 mAbs considerably increased IFN-γ production, whereas culture with control antibodies did not increase IFN- -γ (Figure-5). Blocking 1c3 and 3g2 receptors using mAbs in SEB-activated PBMCs from healthy cows induced IFN-γ production to an average of 4.8 ng/mL.

**Figure-6:**
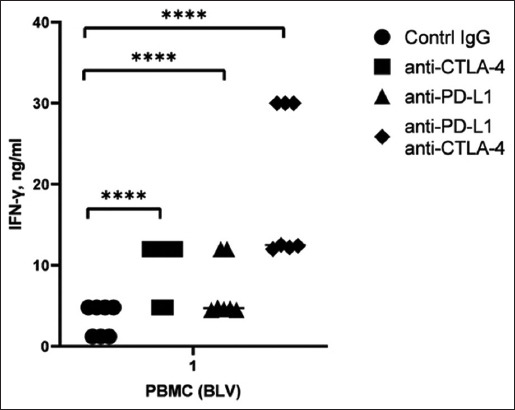
Blocking activities of anti-CTLA-4 and anti-PD-L1 mAbs in PBMCs from BLV+ cattle. CTLA-4=Cytotoxic T-lymphocyte–associated antigen 4, PD-L1=Programmed death ligand 1, BLV=Bovine leukemia virus, mAb=Monoclonal antibody, PMBC=Peripheral blood mononuclear cell. One-way ANOVA was used to identify relationships among samples. Difference in standard deviations p < 0.0001. Changes were considered statistically significant only if the p < 0.001.

Studies on PBMCs from BLV+ cattle have demonstrated that culturing cells with monoclonal antibodies leads to increased concentrations of IFN-γ (Figure-6). Seven PBMC samples from BLV-seropositive cattle were cultured with 25 μg/mL of control IgG and mAbs targeting CTLA-4 and PD-L1 receptors to investigate these effects. When the CTLA-4 receptor was blocked, the induced IFN-γ concentration was 4.8 ng/mL in two samples and 12 ng/mL in five samples. Blocking the PD-L1 receptor also resulted in a range of IFN-γ induction from 4.8 to 12 ng/mL. Simultaneous blocking of CTLA-4 and PD-L1 receptors in all samples, except for one, led to an increase in IFN-γ concentration to 30 ng/mL in three samples and 12 μg/mL in four samples. Statistical comparisons between the untreated and monoclonal antibody-treated groups were conducted using Student’s t-test. Differences between groups were considered statistically significant at probability values of p < 0.001.

## Discussion

Cancer treatments for melanoma and non-small-cell lung cancer involve blocking CTLA-4, PD-1, and PD-L1 receptors [22]. Ipilimumab, a CTLA-4 inhibitor, has gained approval for treating advanced or incurable melanoma. PD-1 inhibitors, such as nivolumab and pembrolizumab, are used for the immunotherapy of metastatic, resistant non-small-cell lung cancer and metastatic melanoma patients. The Food and Drug Administration (FDA) has approved the use of a combination of ipilimumab and nivolumab for metastatic or incurable BRAF WT melanoma patients. In cattle, inhibiting CTLA-4 and PD-L1 increases immune response to infections and vaccine efficacy [2, 10].

These immunotherapies offer controlled side effects and a robust immune response through PD-1/PD-L1 and CTLA-4 blockades. A minority of cancer patients benefit from monotherapy [23]. PD-1/PD-L1 and CTLA-4 blockers used together have shown enhanced antitumor immune responses [22]. The combination of PD-1/PD-L1 and CTLA-4 blockers improves cancer patient survival due to their specific mechanisms.

The study aimed to assess the impact of simultaneous inhibition of CTLA-4 and PD-L1 in BLV+ cattle. The LC-MS/MS analysis confirmed the identity of the produced bovine rCTLA-4 and rPD-L1 proteins based on their peptide spectra matching the sequences of bovine CTLA-4 and PD-L1, respectively. ELISA using commercial anti-CTLA-4 and anti-PD-L1 antibodies confirmed the specificity of rCTLA-4 and rPD-L1. Watari *et al*. [2] obtained recombinant CTLA-4-Ig to study the immunoinhibitory function of CTLA-4 in cattle. It was found that a decrease in the production of IFN-γ in bovine PBMCs was observed following treatment with bovine CTLA-4-Ig preparations. According to this study, drug-induced bovine CTLA-4-Ig binding to the B7 receptor of CD4+ and CD8+ T cells depleted CD8+ T cells. These data suggest that bovine CTLA-4 has immunoinhibitory activity and can be used as an anti-inflammatory drug in cattle [24–27].

To confirm their synergistic effects, 1c3 and 3g2 mAbs were first tested for their ability to increase IFN-γ production in enterotoxin B-activated PBMCs. These experiments confirmed the immunoactivating effects of the resulting mAbs. PBMCs from BLV+ cattle were cultured with 1c3 and 3g2 mAbs to determine the effectiveness of the combined blockade. Combining mAbs increased IFN-γ production in cultured PBMCs from asymptomatic BLV+ cattle. One-way ANOVA demonstrated the significance of differences in mean values (p < 0.0001), confirming the dependence of the increase in IFN on blocking CTLA-4 and PD-L1 receptors. However, in the four PBMC samples from the BLV+ cattle, the combination of anti-CTLA-4 and anti-PD-L1 mAbs did not have a significant activating effect. IFN-γ production in this sample was at the receptor-blocking level of individual mAbs. Perhaps this discrepancy is related to the productivity and physiological characteristics of the animals because the animals used in the study were from the dairy breed of cattle. In addition, hematology blood tests in the four cows revealed a decreased percentage of white blood cells, potentially reflecting fewer cells expressing PD-L1 or CTLA-4. According to Úsuga-Monroy *et al*. [28], PBMCs of BLV+ Holstein cows showed decreased IFN-γ mRNA expression, with the most significant decrease in expression observed in cows with proviral load. There was also a large difference in the expression of IFN-γ by mRNA in PBMC from cows with high proviral loads. In addition to the decrease in the expression of IFN-γ by mRNA, there was a decrease in the expression of interleukins mRNA in BLV+ cows both in PBMCs and somatic cells [29].

## Conclusion

We explored the possibility of treating BLV by targeting bovine CTLA-4 and PD-L1 receptors. LC-MS/MS analysis confirmed the functional similarity of rCTLA-4, rPD-L1, and rPD-L1 proteins to their native counterparts. In BLV-positive cattle PBMCs, recombinant proteins inhibited IFN-γ production. PBMCs from asymptomatic BLV-positive cattle demonstrated enhanced IFN-γ production when treated with anti-CTLA-4 and anti-PD-L1 mAbs. The findings propose that simultaneously targeting bovine CTLA-4 and PD-L1 receptors is a potential therapeutic approach against BLV. A single PBMC sample from a BLV-positive donor did not amplify the synergistic effect. Further investigation with a larger population is necessary to confirm these findings. The studies need to assess a wider array of cytokines.

## Authors’ Contributions

SB and KM: Design and management of the study. KT and ZA: Materials and methods preparation. KT and LT: Generation of genetic constructs and expression and purification of recombinant proteins. ZA: Bovine sera sampling and obtaining PBMCs. SB: Data analysis. KM and KT: Wrote and edited the manuscript. All authors have read, reviewed, and approved the final manuscript.
